# Modulation of tactile perception by Virtual Reality distraction: The role of individual and VR-related factors

**DOI:** 10.1371/journal.pone.0208405

**Published:** 2018-12-03

**Authors:** E. J. Lier, J. Harder, J. M. Oosterman, M. de Vries, H. van Goor

**Affiliations:** 1 Department of Surgery, Radboud University Medical Center, Nijmegen, the Netherlands; 2 Donders Institute for Brain, Cognition and Behaviour, Radboud University, Nijmegen, the Netherlands; Universiteit Twente, NETHERLANDS

## Abstract

**Background:**

Virtual reality (VR) has shown to be an effective distraction method in health care. However, questions remain regarding individual and VR-related factors that may modulate the effect of VR.

**Purpose:**

To explore the effect of VR distraction on tactile perception thresholds in healthy volunteers, in relation to personal characteristics and interactivity of VR applications.

**Methods:**

A randomized three way cross-over study was conducted to investigate the effects of active and passive VR applications in 50 healthy participants. Main outcome measures were monofilament detection thresholds (MDT) and electrical detection thresholds (EDT). Personal characteristics (e.g. age, gender, susceptibility for immersion) and immersion in the VR conditions were analyzed for their effect on VR induced threshold differences.

**Results:**

The use of VR caused a significant increase in both MDT and EDT compared to the control condition (MDT: F (2, 76) = 20.174, *p < 0*.*001*; EDT F (2, 76) = 6.907, *p = 0*.*002*). Furthermore, a significant difference in favour of active VR compared to passive VR was found in MDT (*p = 0*.*012*), but not in EDT. No significant gender effect was found. There was a significant positive correlation between age and active VR distraction (r = 0.333, *p = 0*.*018*). Immersion in the VR world was positively correlated with the effect of VR, whereas visualization and daydreaming were negatively correlated with VR effects.

**Conclusion:**

VR increased tactile perception thresholds, with active VR having the largest effect. Results indicate that the efficacy of VR may increase with increasing age. Gender did not affect VR susceptibility.

## Introduction

Virtual Reality (VR) is a computer technology which creates a realistic experience in a virtual world and immerses the user in a three-dimensional (3D) environment. VR, which is commonly used as a game feature, is increasingly applied in several fields of health care. VR is known to be effective in reducing psychological complaints such as distress and anxiety during painful procedures[1, 2]. Moreover, studies have reported significant pain relief during VR in both acute and chronic pain conditions[3–7].

The underlying mechanism of VR is not yet fully understood, but VR is thought to be effective by distraction, thus diverting attention away from tactile perception. Conscious sensory perception and pain perception require attention, but due to restricted cognitive attention capacity, there is ‘competition for attention’ between distractive stimuli and perceptive stimuli[8–10]. This theory was first described by McCaul and Malott in 1984[11]. The distraction theory suggests that techniques requiring more attentional capacity are more effective than techniques in which less attention is needed [11]. VR is thought to require a large amount of conscious attention since, in contrast to non-VR 2D or 3D games, the user is fully immersed in the virtual world through a Head-Mounted Display (HMD). Therefore, VR might thus provide effective distraction from tactile perception [3, 12, 13].

To date, distraction methods have hardly been studied in relation to tactile perception thresholds. To our knowledge, only two small studies have shown that auditory distraction and music distraction lower tactile perception thresholds[14, 15]. VR distraction has not been studied with regard to tactile perception, however many studies investigated the effect of VR in the context of pain relief. Although VR holds promise for clinical use as a distraction method, several questions remain. First, VR distraction has mostly been studied in specific research populations, such as burn wound patients, children and healthy adolescents[3, 5, 16–21]. It is important to know whether VR is effective in a broad sample of the general (aging) population, as the common hospitalized patient is of an older age nowadays. Second, the influence of individual characteristics on the effect of distraction has hardly been investigated. The influence of personal characteristics such as gender and susceptibility for immersion needs further exploration, as these factors potentially modulate the effect of VR[21, 22]. Knowledge of the role of personal characteristics might be important for a successful implementation of VR distraction in clinical practice, considering the trend towards a more personalized management approach in healthcare.

The main purpose of the present study was to investigate the effect of VR distraction on the detection of tactile and electrical stimuli in healthy volunteers. We aimed to study personal (e.g. age, gender and susceptibility for immersion) and VR (e.g. level of immersion in the VR world) related factors that may modulate the effect of VR distraction, in a study population of all ages. Previous studies showed that distraction modulates tactile perception; here we explored whether distraction through VR applications induces similar effects[14, 15]. We hypothesized that the level of interactivity and immersion in the VR world would correlate positively with a reduction in tactile perception thresholds, supporting the distraction hypothesis[17–19].

## Methods

This was a randomized three way cross-over study conducted at the Radboud university medical center. Participants were stratified based on age (18 to 30 years, 31 to 45 years, 46 to 65 years and >65 years of age, with balanced randomisation). The study was performed according to the principles of the Declaration of Helsinki (2013). The medical ethics committee (CMO Arnhem-Nijmegen) waived formal evaluation of this study according to Dutch law.

### Study population

Eligible participants were healthy volunteers aged 18 years or over. Participants were excluded if they met any of the following criteria: (1) any diseases producing sensory loss (e.g. polyneuropathy, diabetes mellitus, stroke), (2) acute or chronic pain at time of the experiment, (3) use of pain medication or other sensory altering substances (alcohol, drugs) within 24 hours prior to the experiment, and (4) loss of sight or hearing loss, or conditions otherwise impairing effective use of the VR equipment. All subjects provided written informed consent. Participants were not financially compensated.

No reliable sample size calculation could be performed for this study. Fifty participants were included to provide a large enough sample to demonstrate potential differences between VR applications. Withdrawn participants were replaced.

### Interventions

Participants underwent a passive VR condition, an active VR condition and a control condition in a randomised order. Each condition took about five minutes. VR output was generated by a remote computer (Core i5, GTX660Ti, 8 GB RAM). Participants wore a HMD (Oculus Rift Development Kit 2) and stereo headphones.

**Fig 1:**
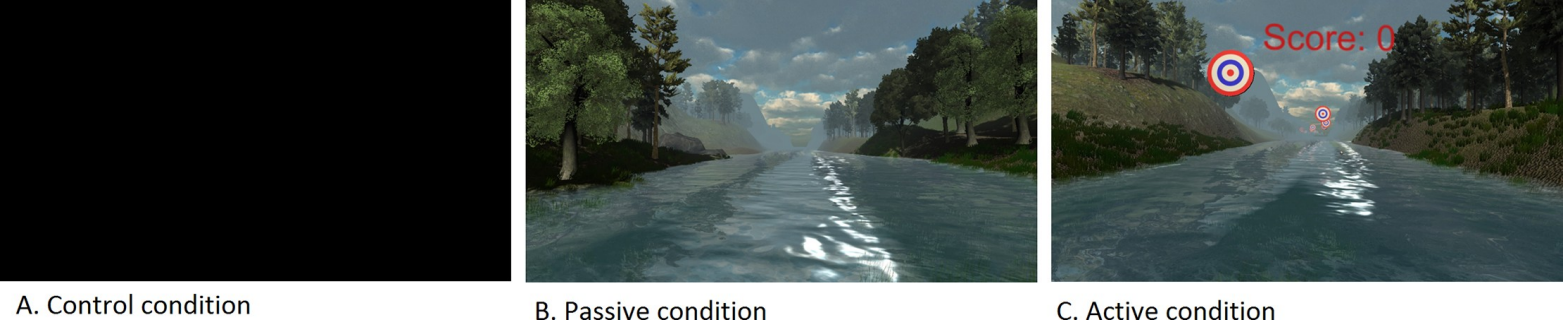
Screenshots of the Virtual Reality (VR) conditions: A = control condition, B = passive condition, C = active condition.

A novel VR application developed by our research group was used during this study (The Virtual River, 2015). In both the passive and the active VR condition, a 360° VR world of a river running through a canyon was composed, in which participants drifted along the river. In the passive condition, calming ambient music and nature sounds were featured. There was no interaction with the VR world. The active condition featured upbeat background music and participants were able to interact with the VR world through specific targets along the route. Participants were instructed to shoot balls as accurately as possible at these targets by slightly tilting their head side to side and up and down. Compared to the passive condition, the speed of the boat was raised in such a way that it would be challenging to hit all targets and get the high score. The control condition included an intervention without audio-visual input, in which participants wore the VR equipment that was not connected to the computer. Screenshots of the VR conditions are shown in Fig 1.

### Outcome measures

Primary outcome measures were monofilament detection thresholds (MDT) and electrical detection thresholds (EDT). Additional analyses were performed on the effects of personal characteristics on both MDT and EDT.

MDTs and EDTs were assessed at random intervals starting 60 seconds after the intervention started. MDTs were measured using six Von Frey filaments, representing a force generated between 1.0 and 32.0 mN. These flexible glassfiber monofilaments are designed and calibrated to bend when a specific amount of force is reached. Since all filaments bend when the specific amount of force is reached, the amount of force given is a function of the test instrument and is not manipulated by the investigator. One stimulus per monofilament was given at approximately 6 cm below the elbow fold on the participant’s right forearm. Participants were instructed to say ‘yes’ on each stimuli they felt. MDTs were measured twice per intervention: once in order of increasing the rigidity and once in a decreasing order. The mean value of these measures was determined as MDT.

The EDT was measured after the monofilament tests. EDT, also known as electrical sensation threshold, was measured using a Quantitative Sensory Testing (QST) device (JNI Biomedical ApS, Denmark) with a ramping current of 0.5 mA/s. Two electrodes were placed side-by-side starting at 3 cm peripheral from the elbow fold on the participant’s right forearm. Subjects were instructed to say ‘yes’ at the moment they felt the electrical stimuli, the value in mA was noted. EDT was measured twice per intervention; the mean value of these measures was used in the analyses.

Personal characteristics were collected using questionnaires. The level of imagination, creativity and (day)dreaming were reported in a custom-made questionnaire using a 5-point Likert scale ranging from *Never* to *Very often*. These items were compiled by our research group as characteristics that might be related to the ability to immerse in a virtual world. Previous gaming or VR experience was registered using yes/no questions (S1 File).

The level of immersion in the VR applications was quantified in a questionnaire with six statements (S2 File), derived from the Dutch version of the Igroup Presence Questionnaire (IPQ)[23].

### Study procedure

Participants were recruited by advertisement. Each participant was given a unique study number based on study entry. Prior to the interventions, subjects were asked to fill in their baseline characteristics and the questionnaire related to the ability to immerse in a virtual world. Participants were made familiar with MDT and EDT measurements. The participants then underwent the interventions in randomized order. A total of six intervention sequences were possible. Afterwards, participants answered 6 questions about the level of immersion in the VR world. This questionnaire served as a wash-out period of 5 minutes between interventions. Any adverse events during the study visit were recorded. All experiments took place in a quiet study environment.

### Statistical analysis

Data analysis was performed using IBM SPSS statistics v22.0. All statistical tests were two-tailed. A *p* value of less than 0.05 was considered to be statistically significant. Descriptive statistics were calculated for baseline characteristics and interventional effects. MDT was transformed on a natural logarithmic scale and EDT was square root transformed to correct for positive skewness, enabling parametric testing. Repeated Measures Analyses Of Variance (RM-ANOVAs) were conducted to test differences in MDTs and EDTs between each VR intervention, using VR conditions as within-subjects variable. Sequence of VR interventions was incorporated as covariate. Exploratory post hoc analyses were performed using parametric tests and Bonferroni correction for pair wise testing. Delta thresholds were calculated for each VR intervention (i.e. Δ Passive VR = passive VR minus control) and outcome variable. Pearson’s correlation tests were performed to examine correlations between the delta thresholds and personal characteristics gathered from the questionnaires. Gender subgroups were compared using independent T-tests on delta thresholds.

## Results

**Fig 2:**
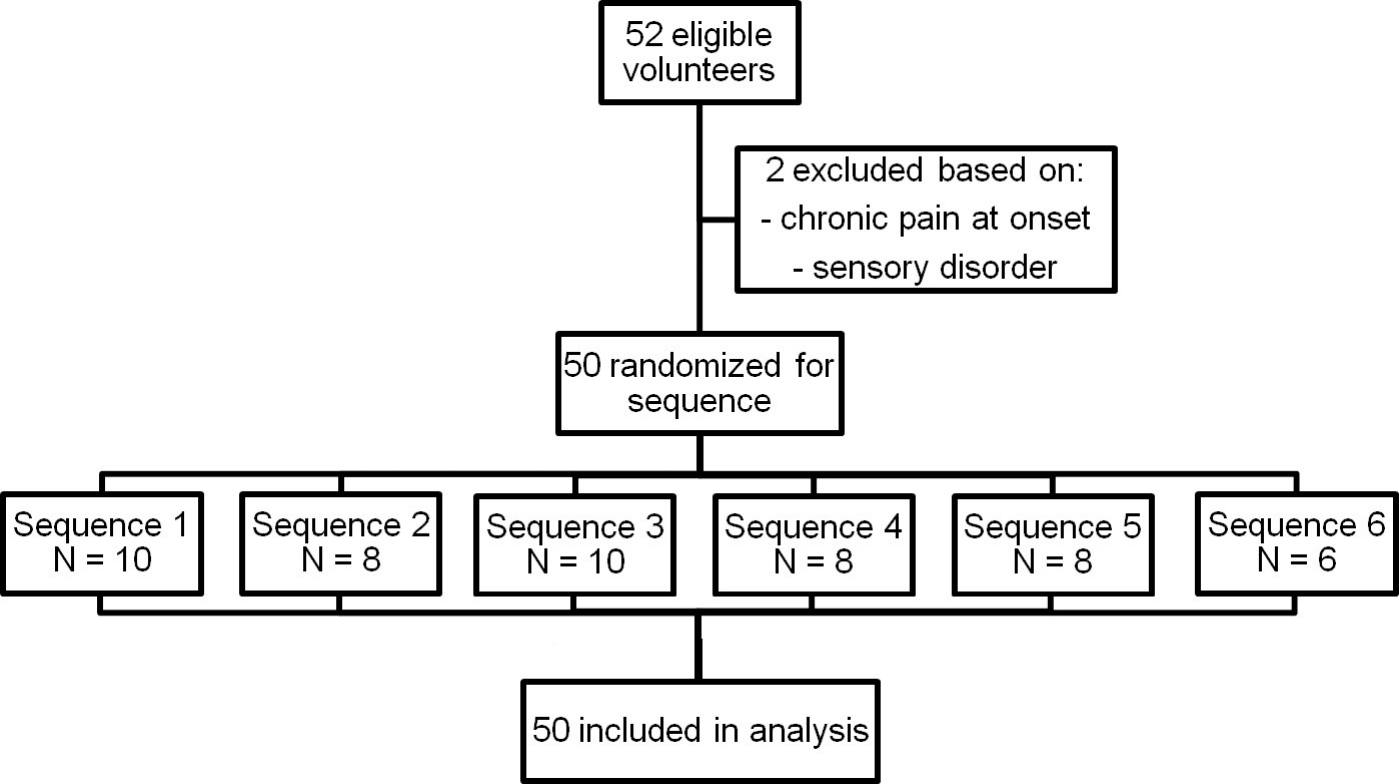
Flow diagram of participants (Conditions: C = control, P = Passive Virtual Reality, A = Active Virtual Reality).

Fifty-two volunteers were screened, of whom two were excluded. Fifty healthy participants, 25 men and 25 women, completed the study protocol and were included in the analyses (Fig 2). Mean age of the participants was 40.9 years (range 19–66).

**Fig 3:**
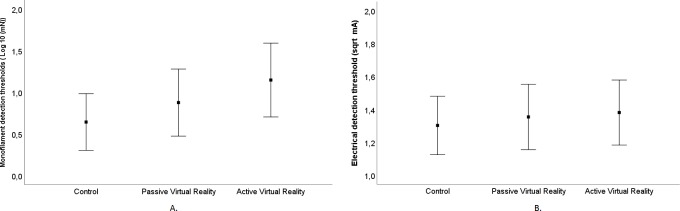
Tactile perception thresholds (mean and SD). A: Monofilament detection thresholds (MDT) after logarithmic transformation, B: Electrical detection thresholds (EDT) after square root transformation.

A significant effect of VR condition was found for both MDT and EDT (MDT: F (2, 76) = 20.174, *p < 0*.*001*; EDT F (2, 76) = 6.907, *p = 0*.*002*). Further post hoc comparison showed that the use of active and passive VR applications caused a significant increase in both MDT and EDT compared to the control condition. Furthermore, a significant difference in favour of active VR compared to passive VR was found in MDT (*p = 0*.*012*), but not in EDT. Fig 3 shows the mean MDTs and EDTs per condition. Sequence of testing was not identified as a confounding variable (MDT: *p > 0*.*2*, EDT: *p > 0*.*6*).

In general, women had a higher sensibility to tactile stimuli compared to men in each intervention including the control setting. Gender did not affect the effect of VR distraction (*p > 0*.*2*) (Table 1).

**Table 1:** Gender differences in monofilament detection thresholds (MDT) and electrical detection thresholds (EDT) (Mean ± SD).

**Condition**	**MDT** **male (n = 25)**	**MDT** **female (n = 25)**	**P value**
Δ Passive Virtual Reality (Log 10 (mN))	0.294 ± 0.264	0.174 ± 0.398	0.214
Δ Active Virtual Reality (Log 10 (mN))	0.531 ± 0.427	0.480 ± 0.499	0.703
**Condition**	**EDT** **male (n = 25)**	**EDT** **female (n = 25)**	**P value**
Δ Passive Virtual Reality (SQRT (mA))	0.052 ± 0.095	0.051 ± 0.128	0.979
Δ Active Virtual Reality (SQRT (mA))	0.080 ± 0.126	0.077 ± 0.120	0.950

**Fig 4:**
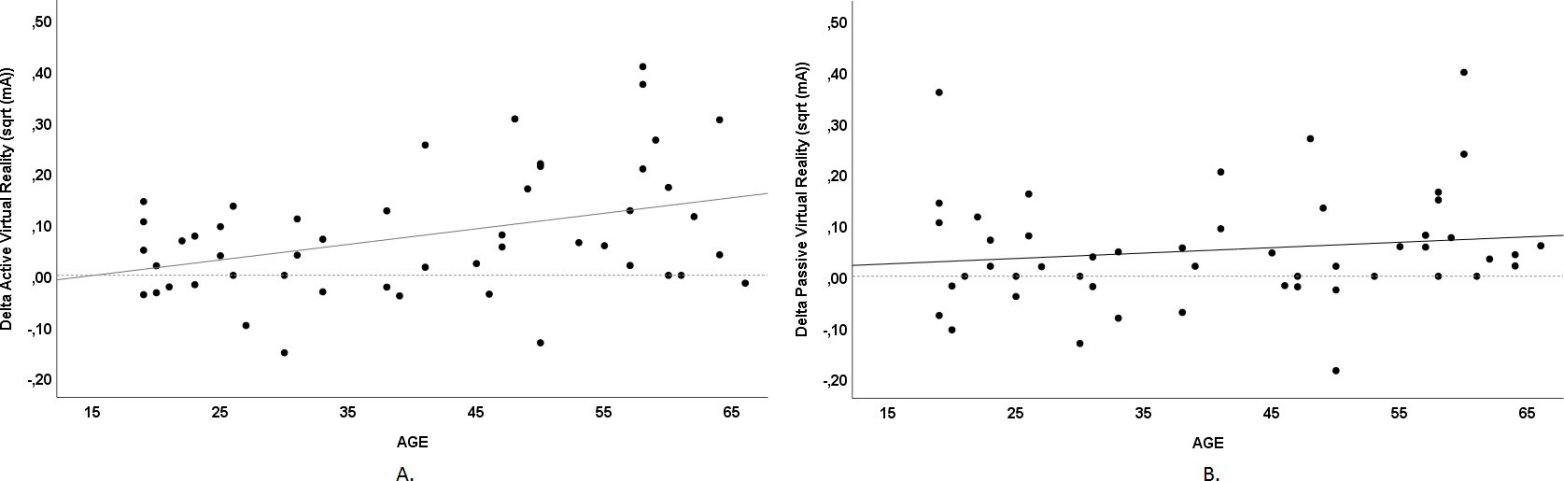
Correlation of age and Virtual Reality in electrical detection thresholds: A. Age and delta Active Virtual Reality, B. Age and delta Passive Virtual Reality.

A significantly positive correlation was found between age and delta VR effect in EDT in the active condition (*r =* 0.386, *p = 0*.*006*), Fig 4A. EDT in the passive VR condition did not significantly correlate with age, but showed similar tendency (Fig 4B).

Three of four self-reported visualization statements and one statement on daydreaming were negatively correlated with passive VR outcomes on EDT (S1 File, easily empathizing, statement 4 *(r = -0*.*307*, *p = 0*.*030)*; visualizing characters in books or films, statement 6 *(r = -0*.*398*, *p = 0*.*004);* visualizing in daily life, statement 7 *(r = -0*.*498*, *p = 0*.*004);* daydreaming, statement 8 *(r = -0*.*379*, *p = 0*.*007*)). These correlations suggest that higher scores on visualization and daydreaming are associated with lower effects of VR on EDT. Other statements on creativity and dreaming were not significantly associated with threshold differences. Previous gaming, VR experience and time spent on gaming did not affect the outcome measures.

Immersion had a positive correlation with an increasing VR effect: the amount of self-reported presence (S2 File, statement 1; *r = 0*.*355*, *p = 0*.*018*) and the amount of immersion in total (S2 File, statement 1–6; *r = 0*.*289*, *p = 0*.*042)* were positively correlated with MDTs in the active VR condition. Furthermore, the amount of self-reported presence in the VR world compared to the real environment, the amount of immersion in the virtual world, and the amount of immersion in total were found to have a significant positive correlation with MDTs in the passive VR condition (Resp. S2 File, statement 2; *r = 0*.*467*, *p = 0*.*001;* S2 File, statement 3; *r = 0*.*333*, *p = 0*.*018;* S2 File, statement 1–6; *r = 0*.*331*, *p = 0*.*019)*. The participant’s awareness of the real environment was not significantly associated with any outcome measure.

Seven adverse events were reported in four participants (aged 20, 22, 39 and 50); one participant felt a slight moment of dizziness after removal of the VR equipment after the passive VR condition; one participant felt disorientated during the active VR condition; one subject reported dizziness in the active and passive VR condition; one subject reported numbness of the fingers/arms in all conditions (exclusion of this participant in data analysis did not change main study outcomes). The adverse events had no consequences for the study procedure.

## Discussion

The aim of the present study was to investigate the effect of VR distraction on tactile perception thresholds and to identify personal and VR related characteristics influencing this effect. This study in healthy volunteers suggests that our in-house developed VR module increases tactile perception thresholds. Both the active and passive VR application increased MDTs and EDTs, compared to the control condition. Furthermore, a significant difference in favour of active VR compared to passive VR was observed in MDTs. Subjectively reported increased immersion in the virtual world was found to have a significant correlation with a decreased tactile perception.

In line with the distraction hypothesis, we found a greater beneficial effect of active VR distraction: when exposed to a more challenging and interactive VR application, participants’ tactile thresholds showed a larger increase compared to the increase in threshold in the passive condition. Although this effect was only significantly observed in the monofilament measurement, the electrical threshold showed a similar tendency. Therefore, by taking into account both measures, we conclude that an active VR application is more distracting. In line with this effect, the subjective level of immersion had a positive correlation with VR effects, although this effect was only found in MDTs, not in EDTs. An explanation for the difference in significant results between MDTs and EDTs could be that EDT might be less sensible for small differences than MDT.

In this study, VR applications were similarly designed for the active and passive VR world. Therefore, the results, favouring active VR compared to passive VR, can be explained by the differences in interactivity and immersion in the virtual worlds, and not by differences in the VR content itself.

In all conditions, women had lower tactile thresholds compared to men, however the effect of VR distraction on tactile thresholds in the passive and active VR condition was not significantly different between genders. Delta EDTs in the passive and active condition were equal in men and women. Delta MDTs in the active condition and even more in the passive condition, were higher in men compared to women, but these differences were not significant. Although Demeter et all[21] found that women experience greater benefits from VR distraction, our results are in line with several studies reporting no gender effect in VR distraction[20, 21, 24, 25].

Furthermore, our data showed a positive correlation between age and (active) VR distraction, suggesting VR being more effective in older persons. This is in contrast to previous VR studies reporting no age-related effects regarding VR susceptibility[19, 24, 25]. Participants in our study had a mean age of about 15 to 20 years higher than most studies, supporting a more robust analysis of age-related effects in VR distraction. The positive correlation found in our study, suggesting an increased VR effect with increasing age, contradict the popular belief that older adults are less susceptible for effects induced by new technologies. No adverse events were reported in participants aged 50 or above. Findings support the use of VR distraction in health care, as the current and future hospitalized patient population is predominantly of an older age.

So far, no study evaluated the effect of personal characteristics (apart from age and gender) affecting VR susceptibility in VR distraction. We studied characteristics including creativity, imagination and dreaming: these characteristics were thought to be related to the ability to immerse, and thus might contribute to an increased analgesic effect of VR. However, we found that the statements on visualization and daydreaming were negatively correlated with VR effects. This might be because our custom-made questionnaire did not measure these characteristics appropriately, or because these characteristics indeed did not contribute to an increased effect on VR analgesics. Alternatively, individuals that use these characteristics might use distraction spontaneously in daily life and might thus be less susceptible to other distraction methods such as VR. VR may be more efficient in people not using immersive strategies, but further research is needed to support or reject this hypothesis.

This study has some limitations. First, both the electrical and mechanical detection thresholds were measured on the participant’s right forearm. The location and sequence of measurements were not alternated. This could have influenced the results because the participants could have been more focused on tactile stimuli at this place. However, the duration of intervals between stimuli varied continuously to decrease the effect of expectancy and habituation. Second, the music and sounds in the applications varied between conditions. The background music during the active condition was upbeat, whereas calming music was featured during the passive condition and the control condition included no sound at all. The music during the active condition was chosen to support the (inter)activeness of the condition and to activate the participants. So we choose for an full experience including matching music. However, we cannot preclude that the music may have played an important role in the observed distracting effects of VR. Final, the current findings pertain to tactile perception and cannot be generalized to other sensory experiences such as pain perception. However, results of our study showing that VR distraction increases tactile perception thresholds are comparable to previously reported effects of VR distraction on pain perception[5, 7, 16, 20, 21]. Furthermore, a greater beneficial effect of active VR distraction compared to passive VR distraction can be seen in both tactile perception and pain perception[17–19]. This suggests that processing of tactile and painful stimuli is in some way comparable. Our study results regarding personal and VR related factors influencing VR susceptibility might thus apply for VR distraction of other sensory modalities as well.

### Conclusion

VR distraction increased tactile perception thresholds, with active VR producing the largest effect. The efficacy of active VR increased with increasing age. Gender did not affect VR susceptibility. Subjectively reported immersion in the VR world was positively correlated with VR distraction effects, whereas reversed effects were found for visualization and daydreaming, suggesting VR distraction was less effective if participants had higher levels of visualization and daydreaming. In working towards a more personalized approach in healthcare, our study results might contribute to further research to identify individuals that will mostly benefit from VR distraction in pain relief. This might include exploration of other personal characteristics related to pain, for example pain coping, anxiety and pain experience.

## Supporting information

S1 File Supporting Information File A: Questionnaire about Characteristics (Creativity, imagination). (DOCX)

S2 File Supporting Information File B: Immersion Questionnaire. (DOCX)
