# Supplementary material for: Modulation of tactile perception by Virtual Reality distraction: The role of individual and VR-related factors
Source: PLoS One. 2018 Dec 3;13(12):e0208405. doi: 10.1371/journal.pone.0208405 (PMC6277112; doi:10.1371/journal.pone.0208405)
Supplement: S1 File — (DOCX) [file pone.0208405.s001.docx]

**Supporting Information File 1: Questionnaire about Characteristics (Creativity, imagination)**

| *Statements* | Never | Rarely | Sometimes | Often | Very often |
| --- | --- | --- | --- | --- | --- |
| 1. *I am a creative person* | □ | □ | □ | □ | □ |
| 1. *I am open to new ideas* | □ | □ | □ | □ | □ |
| 1. *I am open to new experiences* | □ | □ | □ | □ | □ |
| 1. *I can easily empathize with a character, when watching a movie or reading a book.* | □ | □ | □ | □ | □ |
| 1. *I visualize characters in my head when reading a book* | □ | □ | □ | □ | □ |
| 1. *I use empathy to visualize things*   *(e.g. situations in traffic)* | □ | □ | □ | □ | □ |
| 1. *I visualize things that have yet to come (e.g. future meetings)* | □ | □ | □ | □ | □ |
|  |  |  |  |  |  |
| 1. *How often do you have a daydream?* | □ | □ | □ | □ | □ |
| 1. *How often do you dream at night?* | □ | □ | □ | □ | □ |
| 1. *My dreams are lifelike, real.* | □ | □ | □ | □ | □ |
| *Questions* |  |  |  |  |  |
| 1. *Have you ever played 3D games? (e.g. Call of Duty)* | □ No | □ Yes | | | |
| 1. *How many hours a week do you play 3D games (on average)? (e.g. Call of Duty)* | ………………. h / week | | | | |
| 1. *Have you ever played 2D games?  (e.g. Candy Crush)* | □ No | □ Yes | | | |
| 1. *How many hours a week do you play 2D games (on average)?  (e.g. Candy Crush)* | ………………. h / week | | | | |
| 1. *Have you ever experienced Virtual Reality?* 2. *If yes, which device?* | □ No | □ Yes  Device ……………….. | | | |
| 1. *How many hours have you experienced Virtual Reality?* | ………………. h / week | | | | |
