# Supplementary material for: Modulation of tactile perception by Virtual Reality distraction: The role of individual and VR-related factors
Source: PLoS One. 2018 Dec 3;13(12):e0208405. doi: 10.1371/journal.pone.0208405 (PMC6277112; doi:10.1371/journal.pone.0208405)

**Supporting Information File 2: Immersion Questionnaire**

1. In the computer generated world I had a sense of "being there".

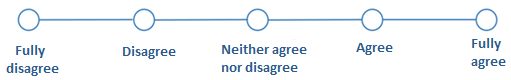

2. I felt present in the virtual world, more than in the real environment.


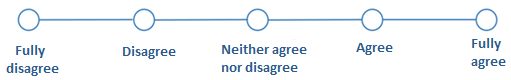


1. I was completely captivated by the virtual world.


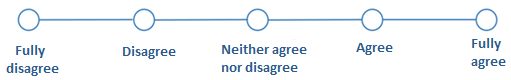


1. I was aware of my real environment.


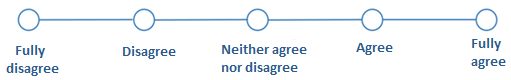


1. I paid more attention to the real environment than to the virtual world.
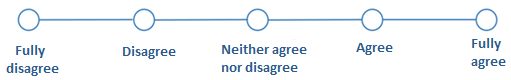

2. The virtual world did not seem real to me.
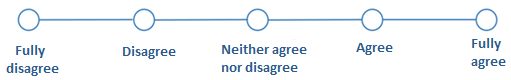

Supplement: S2 File — (DOCX) [file pone.0208405.s002.docx]
